# Can Yoga, Qigong, and Tai Chi Breathing Work Support the Psycho-Immune Homeostasis during and after the COVID-19 Pandemic? A Narrative Review

**DOI:** 10.3390/healthcare10101934

**Published:** 2022-10-02

**Authors:** Bruno Mendo, Mário Gonçalves, Lara Lopes, Luís Carlos Matos, Jorge Machado

**Affiliations:** 1 ICBAS–Institute of Biomedical Sciences Abel Salazar, University of Porto, 4050-313 Porto, Portugal; 2 CBSin–Center of BioSciences in Integrative Health, 4250-105 Porto, Portugal; 3 Faculdade de Engenharia da Universidade do Porto, 4200-465 Porto, Portugal; 4 CTEC–Centro Transdisciplinar de Estudos da Consciência, Universidade Fernando Pessoa, 4249-004 Porto, Portugal; 5 LABIOMEP–Porto Biomechanics Laboratory, University of Porto, 4200-450 Porto, Portugal

**Keywords:** breathing work, homeostasis, Qigong, Tai Chi, yoga, COVID-19

## Abstract

Breathing is crucial in life; nevertheless, the healthcare community often overlooks the health potential of breathing techniques. Conscious manipulation of breathing to achieve specific health goals is found in yoga, Qigong and Tai Chi. This paper reviews the value of breathing exercises as a foremost mechanism for promoting, recuperating and maintaining health. Practices involving breathing techniques are described, and their prophylactic or therapeutic characteristics are explored. The main goals of this review are: (i) to summarize the evidence supporting the hypothesis that breathing practices have a significant beneficial impact on human health; (ii) to provide a deeper understanding of traditional biofeedback practices, particularly yoga, Qigong and Tai Chi, and outline their focus on breathing techniques; (iii) to outline specific immune-related responses, relevant for COVID-19 disorders; and (iv) to call for committed attention and action from the scientific community and health agencies in promoting the implementation of a practical and costless health program based on breathing techniques. This review shows the health potentials of breathing practices and exercises, which, by having a high benefit–cost ratio, could be selected and implemented as a primary standard routine in public health programs.

## 1. Introduction

The concept of “medicine” refers to the science and art dealing with health maintenance in preventing, alleviating, or curing diseases. Medicine can also refer to the strategy to overcome an illness or maintain health [1]. The broad concept of “medical” practices extends beyond the borders tendentiously defined by society, often associated with medical doctors and related professionals (e.g., nurses). Other healthcare practices, commonly referred to as “complementary and alternative medicine” (CAM) [2,3], have been recognized as valuable tools by several Western medical institutions and by the World Health Organization (WHO). Indeed, some have a history longer than conventional medicine (CM). “Traditional, complementary and alternative medicine” (TCAM) is a more appropriate term to describe such traditional therapies globally [4,5]. TCAM approaches emphasize self-empowerment and a holistic view in which life is a union of the body, senses, mind and soul. Thus, the definition of health has been updated to include physical, mental, social and spiritual wellbeing. TCAM covers a broad list of practices, including Qigong, Tai Chi, meditation, mindfulness, and yoga, which could be seen as complementary medical tools positively reported in the literature as useful in the prevention and amelioration of several health conditions, including those related to the respiratory system [6,7,8,9,10,11,12].

Although some of these practices have substantial empirical backgrounds, they have sometimes been ignored in academia and the healthcare community due to a lack of understanding. Perhaps one of the most interesting aspects of TCAM is the simplicity of some practices and the significant effects on health. It is noteworthy that many medical doctors, over time, have been proposing the integration of TCAM practices to improve treatment effectiveness in some CM fields. However, sometimes, the health benefits of TCAM have not been adequately evaluated and considered enough to be accepted as evidence-based [4,5]. A combined political and scientific approach is critical to providing a comprehensive research agenda to establish the potential risks and benefits of TCAM [5]. The present work focuses on a particular aspect that provides a transparent bridge between CM and TCAM inherent in every cell and living organism: breathing. The role of breathing in human physiology and the use of breathing work in the broad field of medicine, especially in the current health crisis, are explored, aiming to provide the reader with the essential information to understand the involved mechanisms and rational applicability of these practices.

According to Damásio (2019), certain physiological conditions and mechanisms generate both spontaneous and provoked feelings and emotions. They reflect movements in simple homeostatic signals that quickly intercommunicate within the extracellular matrix (ECM) and between all cells and compartments of the body [13]. Fromknecht et al. (2013) propose that the nervous system demands a highly rapid and more efficient communication system to promptly achieve homeostatic balance [14]. Indeed, while the nervous system generally operates in the millisecond range, metabolic processes could work in the picosecond range. The fast communication occurring via the extracellular matrix is due to its structural composition. The ECM is rich in proteoglycan and glycosaminoglycan components and hyaluronic acid. This last element is thought to play a critical role because it forms crystals when embedded in water. These crystal conducts promote a high-speed signalling mechanism via proton “jumping” through proton chains without energy expense [14]. Overall, this “communication” hypothesis suggests a swift, holistic and consistent process for maintaining homeostasis based on specific physiological oscillations. Thus, using these “highways”, the effects of breathing techniques on physiology could quickly reach all sections of an organism, exerting significant influence, including on feelings and emotions. Breathing physiology will be further explored to aid the understanding of the ECM hypothesis.

### 1.1. Breathing Biodynamics

Respiration (used here as a synonym of “breathing”) has physiological and psychosomatic implications for human health. Breathing influences how the body produces a supportive molecule that serves as the “energy” for biological functioning. Mitochondrial oxidative phosphorylation, a biochemical mechanism that requires oxygen, is one of the main processes transforming ADP into ATP [15]. The presence of oxygen depends on its inhalation, i.e., breathing. Whether or not this process is well done will reflect changes in the global homeostatic balance in the body.

The biomechanics of lung ventilation coordinate with blood oxygen, carbon dioxide and pH regulation [16]. At the physiological level, shallow and deep breathing are two distinct processes. Deep breaths increase ventilation efficiency via alveolar recruitment and distension, thus reducing alveolar dead space [17], resulting in better distribution and exchange of CO2 for O2 between lungs and blood, increasing arterial oxygen saturation and consequently regulating the internal pH [17,18]. On the other side, during shallow breathing, some parts of the lungs are filled with fresh air and others not, representing less O2 diffusion into the blood circulation. Thus, the organic cells’ proper oxygenation will be missing [19]. Lower oxygen levels in the blood promote, in general, shallow and quick breathing. In this case, a vicious cycle is quickly established, a pattern often associated with anxiety manifestations [20]. It is well known that anxiety and depression are frequently comorbid, with a high prevalence in patients with chronic breathing diseases [21].

Breathing has an important impact on hemodynamics, i.e., blood circulation, and the two systems (cardiovascular and respiratory systems) are often referred to as being intrinsically combined [16,22]. Indeed, a slow respiration rate affects the heart rate and blood pressure, which is related to the resistance of the peripheral vasculature, compliance of the aorta and venous return [23]. It has been reported that deep and slow breathing, around six breaths per minute, induces a significant augmenting venous return [16,24]. A recent study concerning slow-deep respiration in subjects with low initial blood oxygenation showed a significantly greater coupling of respiration and vasomotion with changes in vascular tone (i.e., arteriole diameter), causing oscillations in capillary blood flow [25]. Since the inferior vena cava’s collapsibility occurs during regular inspiration, an efficiency increase of venous return is promoted during slow respiration [26,27]. The modulation of respiration patterns and their effects on venous return has been studied in different scenarios, such as during a single knee extension-flection [24]. Research has shown that different breathing patterns generate distinct cerebrovascular hemodynamic responses [28].

Controlled breathing induces parasympathetic and sympathetic activation, indirectly impacting the cardiovascular, gastrointestinal, respiratory, endocrine, and nervous systems [16,29]. Cardiac parasympathetic efferents are relayed via the vagus nerve inducing cardiac slowing via acetylcholine release. In contrast, sympathetic efferents are relayed via a network of nerves within the thoracic spinal column’s sympathetic chain, accelerating the heart rate via norepinephrine release. These neuro-mediators are released into the sinus nodes, the atrial myocardium and the atrioventricular node [16,30]. The peripheral control of heart and lung movements integrates baroreceptors and chemoreceptors, ventricular and atrial receptors, changes in the respiratory system, the vasomotor system, the renin–angiotensin–aldosterone system and the thermoregulatory system, among others [31,32].

Although both autonomic systems trigger specific answers to the stimulus received, the vagal activity can elicit a much faster influence on the heart, securing a homeostatic background level of control over the heart rate under resting conditions. On the other hand, sympathetic activity, seemingly minimal or absent under resting conditions in healthy humans, is high in several disease states and healthy humans during exercise and physical and mental perturbations [16,33,34].

Studies of diaphragm movement and function show that, for optimal respiration, active control of the diaphragm is required so that during deep and slow inspiration, the abdomen expands instead of the chest, promoting greater lung tidal volume [35,36]. A decrease in respiratory rate alone would lead to hypercapnia (high CO2) or hypoxia (low O2) that, in turn, would trigger the chemoreceptors located in the carotid and aortic bodies, but most notably in the brain stem, inducing a forced increase in respiration rate (hyperventilation) [37,38]. Therefore, to maintain a decreased respiratory rate without disturbing respiratory homeostasis, the tidal volume must be increased via diaphragm movement, preventing the chemoreflex response to hypercapnia and hypoxia [39]. On the other hand, arterial blood pressure monitorization, primarily in the aortic arch and carotid sinuses, involves stretch receptors. The baroreceptor reflex (baroreflex) operates a negative feedback mechanism when in the presence of acute changes via central–neural–autonomic pathways. The baroreceptor activity is reduced when blood pressure is low and increased when blood pressure is high, relaying fast parasympathetic efferent signals via the vagus nerve to the sinoatrial (SA) node to decrease heart rate.

The relationship between heart rate (HR), blood pressure and respiration is known as the cardiorespiratory unit [40], and it depends on the impulses from the autonomic nervous system (ANS). One non-invasive and straightforward ANS activity measure is evaluating heart rate variability (HRV), which is a relevant quantitative marker [30]. The HRV refers to the time variability between consecutive heartbeats (RR interval) and consecutive instantaneous heart rates [16,38]. Lower HRV is frequently an indicator of abnormal and insufficient adaptation of the ANS, provoking poor physiological function, while higher HRV is a signal of good adaptation and characterizes a healthy person with efficient autonomic mechanisms. Hence, HRV allows assessing the ANS balance under physiological conditions such as wakefulness and sleep, different body positions, physical training, and pathological conditions [16,30]. The baroreceptors are tightly connected to the low cardiac frequency and HRV oscillations predominantly accountable for these responses [16].

The synchronicity between HRV and the respiration phases is called respiratory sinus arrhythmia (RSA), which indicates that R–R intervals are lengthened (the heart rate decreases) during expiration and shortened (the heart rate increases) during inspiration [16,41,42]. Hayano and Yasuma (2003) suggested that RSA could be an intrinsic function for improving pulmonary gas exchange efficiency during the cardiorespiratory system’s resting state, resulting in minimum energy expenditure [43]. This hypothesis is supported by increased RSA during sleep (in a supine position), with slow, deep respiration and relaxation, but it is reduced during exercise and anxiety states. According to Billman (2011), arterial blood pressure increases during expiration and decreases during inspiration under steady-state conditions [44].

Interestingly, this physiological behavior can influence controlled, slow respiration, which increases baroreflex sensitivity and HRV amplitude [45] and significantly decreases mean blood pressure [16].

Thus, based on all the aforementioned physiological mechanisms, it is clear that breathing can substantially affect the entire biological system, with acute consequences on people’s health status. Stress is one of the primary agents of disease in today’s society [45]. Can the practice of specific breathing patterns contribute to preventing, for instance, chronic conditions or psychological distress? Can it have a positive impact on already existing pathologies? Those are two of the many questions demanding research, which encouraged the present review.

### 1.2. Traditional and Complementary Medicines

#### 1.2.1. Traditional Chinese Medicine

Traditional Chinese medicine (TCM), dating back 2000 to 3500 years, was developed in China based on traditional philosophy and culture [46,47]. It comprises herbal medicine, dietary therapy, acupuncture and moxibustion, manual therapy (Tuina), and traditional biofeedback exercises (Qigong and Tai Chi). This diversity of therapeutic methods makes TCM one of the most relevant systems of TCAM, playing a pivotal role in the framework of integrative medicine [47,48,49,50]. Chinese medicine doctrines integrating notions such as the yin and yang and the five evolutive phases are rooted in books such as The Yellow Emperor’s Inner Canon and Yi Ching. Starting in the 1950s, in the People’s Republic of China, these precepts were standardized by the Chinese government into a systemized TCM, attempting to integrate it with modern scientific medicine concepts based on physiology, anatomy and pathology [51]. However, since most terminologies in classic TCM texts originated from Chinese philosophy, some believe that to understand the physiology of TCM, one should have some knowledge of Chinese philosophy [46,47].

TCM recognizes the human body as a system and adopts a cybernetic approach. Thus, it can be characterized as holistic, emphasizing the human body’s integrity and the close relationship between a person and their social and natural environment. TCM focuses on health maintenance, enhancing the body’s resistance to diseases and treating infirmity [46,47]. Chinese doctors look for all active expressions of life, indications of a balance of energies that flow in a cyclic sequence along the meridians (conducts), forming an interconnected network that promotes whole intrinsic homeostasis. This movement and interchange among the five elements (wood, fire, earth, metal and water) express the human body’s physiology [31]. According to a correspondence system, the findings’ organization allows identifying these functional systems (Zang Fu) [46]. A modern interpretation of Chinese medicine may be defined as “a system of sensations and clinical signs and findings designed to define the regulatory state of the body” [52]. According to Porkert and Hempen (1995), TCM is primarily concerned with the function, movement and vital manifestations that characterize every pathological abnormality [46]. “Agents” are defined as factors that elicit a functional deviation, and these can be exogenous (climatic or social), endogenous (emotional or constitutional factors favoring a variation from the normal function) or neutral [50].

Conventional medicine explores the specificity of morbidity. However, only targeting the specificity is not enough to stop morbidity progress since the non-specificity can sometimes influence or change the process [53]. On the other hand, TCM mainly explores the reality of morbidity by checking the external appearance. Following the “Zheng” theory [47], different diseases may be treated using the same therapeutic approach if they exhibit the same symptoms. However, the same condition may be treated by other therapeutic methods in different patients or the same patient in the presence of different symptoms.

Among TCM therapeutics, Qigong and Tai Chi are the most suitable for relaxation and rehabilitation, contributing significantly to homeostatic and neurovegetative regulation. These practices often use slow breathing with closely related health outcomes [54].

#### 1.2.2. Ayurveda Medicine

At this point, it makes sense to mention Ayurveda, the traditional Indian medicine (TIM), which, together with traditional Chinese medicine (TCM), represents the most ancient traditions of medicine. The Sanskrit word Ayurveda means “the science of life”. Ayurvedic knowledge originated in India more than 5000 years ago and is often called the “mother of all healing”. It stemmed from the ancient Vedic culture and was passed from accomplished masters to their disciples for thousands of years.

Ayurveda practices aim to help persons achieve a balanced body, mind and consciousness, making lifestyle changes to bring and maintain homeostasis. Just as everyone has a unique fingerprint, each person has a particular energy pattern, an individual combination of physical, mental and emotional characteristics, which comprises their constitution. According to Ayurveda, life is conceived as the union of the body, senses, mind and spirit, known as Prakriti or individual nature, which has a central role in Ayurveda therapeutics [55,56,57].

According to Ayurvedic philosophy, the entire cosmos (the material universe at all scales of life) includes the energies of five elements known as “pancha mahabhutas”. Those include “akasha” (ether/space), “vayu” (air), “teja” (fire), “aap” (water) and “prithvi” (earth). Energy is required to create movement so that fluids and nutrients reach the cells, enabling the body’s functioning. Energy is also required to metabolize the nutrients in the cells and lubricate and maintain their structure. Therefore, in biological systems such as humans, elements are structured into three forces (energies), which govern all life processes. These three forces (“kapha”, “pitta” and “vata”) are known as the three “doshas” or merely the “tridosha” [55,57]. “Vata” has the mobility and quickness of space and air (movement); “pitta” is the digestion or metabolic qualities of fire; “kapha” is the stability and solidity (lubrication and structure) of water and earth. “Vata”, “pitta” and “kapha” are combinations and permutations of the five elements that manifest as patterns in all creation. The “tridosha” regulates every physiological and psychological process. The interplay among them determines the qualities and conditions of each individual. A harmonious state of the three doshas creates balance and health; an imbalance, which might be an excess (“vriddhi”) or deficiency (“kshaya”), manifests as a sign or symptom of disease [55,56,58,59,60]:

- “Vata” governs breathing, blinking, muscle and tissue movement, pulsation of the heart, and all movements in the cytoplasm and cell membranes. In balance, “vata” promotes creativity and flexibility. Out of balance, “vata” produces fear and anxiety.
- “Pitta” governs digestion, absorption, assimilation, nutrition, metabolism and body temperature. In balance, “pitta” promotes understanding and intelligence. Out of balance, “pitta” arouses anger, hatred and jealousy.
- “Kapha” forms the body’s structure, such as bones, muscles, and tendons, and provides the “glue” that holds the cells together. “Kapha” supplies the water for all bodily parts and systems. It lubricates joints, moisturizes the skin and maintains immunity. In balance, “kapha” is expressed as love, calmness and forgiveness. Out of balance, it leads to attachment, greed and envy.

These practices involve the concept of “life force energy”, which Yogis call “Prana”, Chinese healers call “chi” or “qi”, and Freud called libido. This energy is experienced in exciting or scary situations. Those situations create a sensation often reported as a vital charge that flows through, opposite to the absence of this sensation in depressed or exhausted states. Due to psychological wounding, most persons have created blockages that mean they avoid experiencing their life force’s full flow. The power to direct the energy flow is innate. Each person may increase or decrease the energy levels needed to create, heal, awaken, or inspire. The energy center is called a “Chakra”, meaning “wheel or disk” in Sanskrit. The word’s origin stems from how the energy spins from one energy center to the next in the network with the body’s physiological systems. In total, seven centers (“chakras”) are located from the base of the spine to the crown of the head: root; sacral; solar plexus; heart; throat; third eye and crown. Each plays a role in controlling emotions and connecting to surroundings [56,61,62]. Chakras are idealized as swirling wheels of energy where matter and consciousness meet. The healing energy (“prana”) is understood as a vital life force responsible for keeping the person vibrant, healthy and alive.

Both internal and external factors disturb this “energy” balance, causing effects on each person’s constitution that change from a balanced to an unbalanced state. These factors stressing balance include the emotional state, diet and food choices, seasons and weather, physical trauma, and work and family relationships. Once these factors are understood, appropriate actions can be taken to nullify or minimize their effects, eliminate the causes of imbalance, and reestablish the original constitution. Within the body, two primary states can be found, the ordered, which manifests balance and health, and the disordered, which manifests imbalance and disease. While there is a constant interaction between order and disorder, understanding the nature and structure of disorder helps reestablish order [55,56,60].

Ayurveda has various techniques for assessing health during diagnosis. The practitioner carefully evaluates critical signs and symptoms of illness, especially concerning the origin and cause of an imbalance. During direct questioning, observation and a physical exam, several techniques are used, such as taking the pulse, observing the tongue, eyes and physical form, and listening to the voice’s tone. Self-care and treatment recommendations may include implementing lifestyle changes, starting and maintaining a suggested diet, use of herbs and yoga’s deep breathing exercises. In some cases, a cleansing program called panchakarma is recommended to help the body rid itself of accumulated toxins to benefit from the various suggested treatment procedures [55,56,57,60]. Ayurveda lags far behind scientific evidence in the quantity and quality of randomized controlled clinical trials (RCTs) and systematic reviews. However, yoga and its breathing techniques have gathered significant scientific relevance in recent decades.

## 2. Methods—Literature Search

This study was designed as an overview to provide new insights on using simple breathing techniques to tackle global health problems, including the current COVID-19 pandemic, which mainly affects respiratory function. A “quantitative analysis” section was also included, which will be further described.

**Figure 1:**
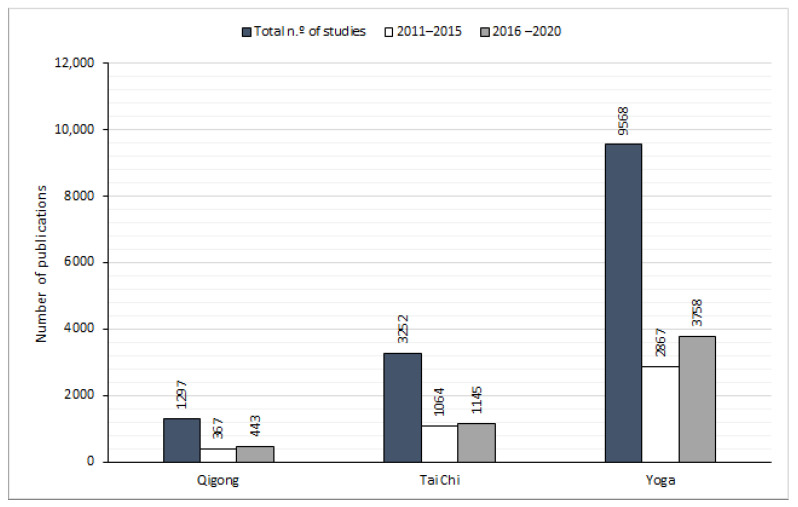
Graphical representation of the analytical data collected from the literature search showing the total number of publications per traditional heath practice (source Web of Science).

For the quantitative analysis regarding the different TCAMs selected, a literature search was conducted in Scopus, Web of Knowledge, Pubmed and Psychinfo (databases). Searches were conducted during 2020, on March 14, on May 4, and again on July 26. From this search, the following information was considered: the date of earliest publication, the number of total studies, the number of studies from the last five years (2020–2016) and the number of studies from the previous five years (2015–2011). The idea with these last two steps was to determine whether the work being done on these subjects is increasing or decreasing. The database with more reported studies was selected to conduct this analysis (this analysis can be found in Figure 1). Scopus was the database with more indexed articles for all the keywords. For each of the TCAMs selected, the same keywords were used for the four database searches, as follows:

- Qigong—“Qigong”, “Qi Gong”, “Chi Kung”, “Qi kung” and “Chi Gong”;
- Tai Chi—“Tai Chi” (some studies were excluded from the count given that they referred to Tai Chi T’ao, a journalist with particular socio-political relevance);
- Yoga—“Yoga”.

The results are summarized in Figure 1. We also extracted relevant qualitative information from the literature to analyze the previously mentioned systems, breathing physiology, TCM and TIM’s theoretical background. Both quantitative and qualitative analyses are presented ahead.

## 3. Results

### 3.1. Quantitative Analysis

According to the analysis on Scopus, the scientific study of yoga started as early as 1904 and showed a total of 9568 papers. Receiving less but still significant scientific attention, Tai Chi follows with a total of 3252 papers, the first article dating back to 1967. These two are followed by Qigong, whose first entry was in 1981, with a total of 1297 papers. Additionally, in two recent periods (2011 to 2015 and 2016 to 2020), a brief analysis shows a recent evolution of interest in those topics, with an outcome of 2867 and 3758 for yoga, 1064 and 1145 for Tai Chi, 367 and 443 for Qigong, respectively.

Qigong was notably less investigated, which might be due to confusion with Tai Chi. Future studies should make a clear distinction to analyze the literature correctly. Zhang et al. (2020) [63] suggest that Qigong and Tai Chi’s benefits are diverse and inconsistent in the literature.

### 3.2. Qualitative Analyses Based on Review—General Reported Effects

#### 3.2.1. Conventional Medicine

Although there seems to be no vast literature regarding respiration as a therapeutic technique in and of itself, it is common to find it as non-prominent means among others. For example, many elements are considered inside the standard delivery room when pregnant women labor. In optimal conditions, the professionals need to be ready to perform a series of interventions on demand, such as caesarean section and administering epidural analgesia [64,65]. However even though this is important, there is always something asked of every woman during labor: “breathe!”. Indeed, breath control has been shown to work as pain relief and it decreases the caesarean section intervention rate [66], which is associated with a higher level of satisfaction regarding the birth experience. It can be hypothesized that this higher satisfaction and acquisition of breathing skills can then impact the post-labor life of mothers. Moreover, a simple daily 5 min breathing exercise applied throughout 30 sessions or a three-day intervention (with exercises three times per day) has significantly reduced anxiety in pregnant women [67,68].

Diaphragmatic breathing could be an effective and cheap solution to overcome the problems associated with rumination syndrome (RS), one of the least understood functional gastroenterology disorders [69]. As an example, an 18-year-old man who did not respond to repeated trials of acid-blocking medication or metoclopramide to prevent severe gastro-esophageal reflux (GER syndrome, the original diagnosis) was advised to consult a surgeon to undergo a Nissen fundoplication, a surgical process in which the top part of the stomach, the “fundus”, is stitched around the esophagus to aid its closing. The clinician taught him how to do diaphragmatic breathing in the visit, and after two months and two consultations with a behavioral psychologist, remarkable improvements in regurgitation symptoms were experienced [69]. Diaphragmatic breathing has been shown to significantly reduce cortisol levels and negative affect, i.e., the experience of negative emotions [70]. One study tested the hypothesis that biofeedback-assisted diaphragmatic breathing, along with systematic relaxation, would be more effective than propranolol in the long-term prophylaxis of migraine [71]. This collaborative practice was demonstrated to be more effective in the long-term treatment of migraines. In that study, the effects of this specific breathing technique were not disassociated from relaxation, as relaxation is undoubtedly one of the many positive effects of these breathing techniques. Moreover, when no contraindicating medical conditions are present, hyperventilation can be used as a therapeutic method to treat anxiety and as a diagnostic tool [72]. As a technique that uses, amongst other means, hyperventilation, holotropic breathing has been proposed to be an alternative therapeutic procedure for some psychiatric conditions [73].

Herbert Benson, M.D., from Harvard Medical School, developed a relaxation response method that is primarily a breathing pattern maintained from 10 to 20 min once or twice a day [74].

This review defines slow breathing as any rate from 4 to 10 breaths per minute (0.07–0.16 Hz). A typical human respiratory rate is 10–20 breaths per minute (0.16–0.33 Hz). It is regarded as a method to improve health, with application in diverse conditions, such as the alleviation of premenstrual syndrome symptoms [75] or decreased blood pressure in those cases where an individual’s high blood pressure is caused by stress [76], to name a few.

Deep abdominal breathing is a crucial component of diverse, widely used psychotherapies, significantly reducing stress, anxiety and depression. Systematic Muscular Relaxation by Jacobson and Systematic Desensitization Programs by Wolpe are two clear examples of breathing as an essential part of the therapeutic procedure to manage psychosomatic distress and phobias [77,78].

The benefits of having an active lifestyle are widely known [79]. Physical activity can substantially improve health by reducing the risks of dying prematurely from heart disease, developing diabetes and high blood pressure, and reducing feelings of depression and anxiety. An amelioration of the cardiorespiratory system is frequently described [80], but the focus rarely rests on the respiratory part alone. More research should be performed to clarify how the health changes associated with sports are affected by the breathing patterns employed during physical activity. If a significant contribution of breathing is proved systematically, breathing techniques could be a generic alternative to prevent disease, with specific interest for those with physical disabilities. It is noteworthy that regular physical activity is one of the primary measures to prevent obesity [80], a condition associated with a higher risk of developing multiple other illnesses [81]. Some studies show that obesity harms respiration, causing impairment in breathing patterns and respiratory mechanisms expressed by shallow breathing, eventually leading to ventilatory failure [82,83].

Breathing exercises and practices have always been used in CM, although not often as a primary therapy. Scientific research has shown the effects of breathing on several health scenarios, such as augmenting immune cell count, enhancing the immune response under stress conditions [84,85], improving parasympathetic activation and reducing sympathetic tone with impacts on blood pressure, hormones and general stress levels [85,86,87,88,89]. Although breathing exercises have been shown to produce positive health outcomes, CM professionals rarely educate their patients to breathe correctly, or promote relaxation response exercises, as they do for simple everyday activities such as personal hygiene, sleep, exercise or diet. This happens probably because most professionals are still unaware of its importance as there is no official recommendation for these practices. There is no public health program promoting good breathing practices because public health policy in Western countries is still anchored on CM grounds where breathing has still not been tested by prominent official authorities as a valued therapy. Fortunately, this scenario has changed in recent decades and there is more recognition and integration of CAM practices. CM professionals have been seeking alternative or complementary approaches to compensate for the side effects of pharmacological interventions.

#### 3.2.2. Qigong Breathing Effects

Many historical forms of Qigong are used for general health enhancement purposes, and some are recommended for specific TCM diagnostic categories. This practice incorporates many simple repetitive movements or postures (standing or sitting; inspired by nature) [54]. Qigong is a process in which “qi” is understood as the vital energy or a functional capacity for a tissue or organ to work, also interpreted as “life force” in some schools of TCM, and “gong” is the activation or training [90]. This energy (“qi”) is achieved through breath control and a “special state of mental awareness”, resulting in enhanced homeostasis (mechanism of vegetative regulation) [54]. Another designation would be the one of “ancient oriental mindful exercise” [91], which can be understood as an exercise focusing on self-awareness and intrapersonal mind–body alignment to be accompanied by low-to-moderate muscular exercise and non-judgmental meditation. It differs from most physical activities centered on kinesthetic training and is relatively disconnected from the mind or meditation.

During Qigong practice, a relaxed concentration state must be achieved, avoiding energy loss in multiple thoughts [90]. The forebrain and midbrain are particularly active in this meditation, and high production of both beta (attention to external filter) and theta (interoception sense) brain waves are registered [92]. The body’s energy effect may be measured as heat through infrared thermography [93] or by changes in skin electrical potential of acupoints [94,95]. This might indicate higher energy and conductance deep in the tissue between different acupoints, as predicted by TCM theory [96,97,98]. These acupoints are interconnected, forming a functional structure designated by the aforementioned “meridians” or conducts, presenting low electrical impedance and high conductance. These acupoint conductance changes suggest that their implications for generating symptoms, syndromes and diseases are valid [96,97,99,100]. Matos et al. (2017, 2020) demonstrated that a focused mind changes the molecular vibrational state of water samples reflected by the responsive magnetic field strength, radiation, pH and electrical conductance measurements [101,102]. Furthermore, and relevant to the homeostatic process, a close relationship is suggested between blood and electrical flow in response to these (breathing) exercises [92]. These effects can be achieved through specific Qigong techniques, such as “White-Ball” in the Heidelberg model of TCM [93,103]. Qigong practice benefits have been shown on different components of wellbeing, such as cognitive behavior, distraction, social interaction, cardiovascular fitness, amine regulation and endorphin regulation [91,104,105,106]. This practice generally provides a non-pharmacological modality for achieving biopsychosocial health for those suffering from anxiety, stress, depression, symptoms, chronic pain, immunity, infection and reduced quality of life [91,106,107,108,109,110].

Qigong has been proven to positively affect sleep quality, anxiety and depression [111,112,113]. Indeed, compared to control groups, participants practicing Qigong registered better sleep quality [111] and significant decreases in anxiety and depression when compared with active [88,114] and inactive controls [115], or when comparing before and after the qigong exercise [90]. Young flutists submitted to a seven-week Qigong training demonstrated lasting effectiveness in reducing anxiety-related manifestations [103]. This suggests that new vegetative patterns have been created by the Qigong practice and that, eventually, a sharper awareness of one’s body sensations was developed. This hypothesis being correct, participants would not only have a new repertoire of responses but would be accustomed to changes in bodily sensations, not engaging in the widely known process of catastrophizing sensations such as panic attacks typical in anxiety disorders [116]. Not reacting automatically to changing body sensations may be vital in having more choice over how we feel.

Current research suggests a favorable effect on bone health for those practicing Qigong or Tai Chi, reporting slower bone loss and fewer fractures [54]. Compared to no-exercise controls, women following Qigong exercises presented an increased bone mineral density [54]. One of the most consistently reported findings is the significant reduction in blood pressure reported in multiple studies, especially when Qigong was compared to inactive control groups or waitlist controls [88,117]. Reduced heart rate is also reported as well as increases in heart rate variability which, together with blood pressure reduction, suggest that one or several of the critical components of Qigong (e.g., breathing, concentration, light movement) may affect sympathetic and parasympathetic balance and activity [54,87,88,90,118]. Improvements in heart-health biomarkers, such as lipid profiles [87] and serum B-type natriuretic peptide levels [119], were shown in response to Qigong and/or Tai Chi compared to inactive or usual care controls, respectively. Participants with a history of chronic atrial fibrillation [120] or heart failure [119,121] and women treated for breast cancer [122] achieved significant improvements in the walk test following Qigong or a combined Qigong/Tai Chi intervention compared to an inactive control, or a psychosocial group-supported control intervention.

Physical function measured with a wide variety of performance indicators, such as balance, strength, flexibility and handgrip, showed significant improvements with both Qigong and Tai Chi exercises for sedentary elderly healthy adults or those deemed at risk for falls compared to a control intervention or waitlist control [122,123,124,125,126]. Qigong exercises may reduce the symptoms of Parkinson’s disease and improve the body functions of patients in both the mild and moderate stages [127]. Furthermore, it may promote an improvement in healthy physical behavior, self-control, sociability and sensory and cognitive awareness in behavioral and autism disorders [106,128]. Compared to inactive control groups, Qigong practice enhanced self-efficacy and the ability to handle stress and novel experiences in patients with perceived symptoms (without a diagnosis) [115,129]. Self-reported neck pain and disability [130] and fibromyalgia symptoms [131] improved slightly with Qigong compared to other exercise interventions or usual care control.

A study examining blood markers related to stress response, i.e., norepinephrine, epinephrine, and cortisol levels, significantly decreased in response to Qigong compared to a waitlist control group [88]. Concerning immune-related responses, improvements in several immune-related blood markers, including the total number of leukocytes, eosinophils and percentage of monocytes, and a significant increase in antibody levels in response to flu vaccinations, have been reported in people practicing Qigong, compared to usual care [132,133]. Interleukin-6, an important marker of inflammation, was significantly modulated in response to Qigong compared to a no-exercise control group [134]. According to Oh et al. (2020), current evidence indicates that Qigong practice has a physiologic impact on immune system functioning and inflammatory responses [133].

Lastly, concerning the dramatic global situation related to COVID-19, Zhang et al. (2020) proposed a trial study involving a Qigong program to evaluate the efficacy of physical and mental rehabilitation of patients with COVID-19 [63]. Considering that patients with COVID-19 may suffer from panic and anxiety motivated by the overall mortality and infectivity, the researchers suggested combining abdominal breathing and lip breathing to produce six different sounds (xu, he, hu, si, chui and xi) and low-intensity body movements. This breathing pattern can change the dysfunctional rapid and shallow breathing pattern, extend the trachea’s opening time and maintain the patient’s airway pressure within a physiological range, thereby improving gas exchange [135,136]. Clear benefits on the psychophysiological health of patients with severe pneumonia are expected with this specific breathing.

In summary, there are many positive effects of Qigong therapy, such as reducing stress, fatigue, anxiety and depression, improving sleep quality and psychophysiological disorders associated with balance and stability in older people, relieving pain in knee osteoarthritis, fibromyalgia and chronic neck and back pain, hypertension decrease, cardiovascular and pulmonary benefits, boosting the immune system and related responses, and management of cancer, behavioral and autism disorders. Qigong mind–body functions result from regulating the global homeostatic vegetative network [137].

Breath control may be responsible for many of the effects reported above. Regardless of characteristics, breathing is an essential (if not the most important) part of Qigong, which is, as has been mentioned, one of the primary therapies of TCM. Breathing has been an essential concept in therapy or disease prevention, in which the systematic practice of specific breathing patterns, such as those used in Qigong, has been proven to produce positive outcomes. Indeed, TCM practitioners often advocate that “two of the most important things to maintaining health are good sleep and good breathing”.

#### 3.2.3. Tai Chi Breathing Effects

Tai Chi has been used for centuries as a traditional Chinese wellness practice sharing theoretical roots with Qigong [138]. According to Jahnke et al. (2010), Tai Chi represents an expansive philosophical and theoretical notion that describes the natural world (i.e., the universe), a dynamic interactive balance of light and dark, movement and stillness, waves and particles. It is typically performed as a highly choreographed, lengthy, complex combination of deep diaphragmatic breathing and relaxation, with a harmonious flow of movement [139]. When regarded as a meditative movement, it is intended to promote internal functional balance to neutralize stress, increase longevity and increase personal tranquility [54,106]. As can be strongly emphasized, breathing is a fundamental element of this practice.

Similar to Qigong, post-menopausal women practicing Tai Chi presented bone loss delay and fewer fractures than usual care [140]. Compared to inactive control groups, subjects with arthritis experienced improved self-efficacy [141], and fibromyalgia patients experienced improvements in managing pain [142] after practicing Tai Chi. Significant benefits on heart rate [141,142], heart rate variability [143], blood pressure [144], heart failure [119,145], breast cancer [122], performance indicators of physical function (falls and balance) [143,146], quality of life and self-efficacy [147] and lipid profiles and serum B-type natriuretic peptide levels [119] have been recorded. Perceived symptoms of heart failure disability [148], sickness impact scores [149] and sleep quality [150] decreased in response to Tai Chi practice. Reduced depression, anxiety, and stress among patients with osteoarthritis [151], as well as significant improvements in adrenaline, heart rate, noradrenaline and cortisol levels with Tai Chi practice [54,152] have been reported. Moreover, in response to the vaccine, varicella-zoster virus titers and T-cells increased significantly more among Tai Chi practitioners than waitlist controls [153]. Furthermore, improvements in thyroid-stimulating hormone, follicle-stimulating hormone, triiodothyronine [54] and lymphocyte production [154] have been reported in response to Tai Chi practice compared to matched controls. Pre–post Tai Chi intervention designs have also shown an improvement in immunoglobulin G (IgG) and natural killer (NK) cells [155].

Evidence supporting Tai Chi’s beneficial effects on health has been growing in the last decades. These include reduction in blood pressure [144], maintenance of bone density [156], increased balance, augmented quality of life [145,157] and improvement in depression symptoms [158,159] and in immunoglobulin G [160] levels of immune cells and natural killer cells [133,155]. Most of the previously mentioned studies used a relatively short-term and simplified practice. However, it is noteworthy that Tai Chi, like Qigong, requires continuous, everyday practice [54].

With a few exceptions, most studies indicate that Qigong and Tai Chi have great potential for improving health and general quality of life in healthy and chronically ill patients, including those with cancer, post-stroke conditions, arthritis, and muscular dystrophy, to mention a few. Although the evidence regarding TCM breathing techniques suggests health benefits, more (and systematic) studies need to be conducted on Qigong and Tai Chi techniques to examine the degree and specificity of benefits concerning several symptoms or diseases. Considering that part of the benefits may be associated with aerobic exercise, these studies can further clarify the potential of Tai Chi and Qigong compared to regular exercises.

#### 3.2.4. Yoga Breathing Effects

Yoga is an ancient Hindu practice part of Ayurveda medicine. It dates back more than 5000 years and integrates mental and physical exercises to attain a particular state of being—Samadhi (sanscrit “sama”, which means together, and “dhi” which means mind). The most straightforward way to translate Samadhi is to say that it is a “complete state of concentration”, however, for yogic practitioners, it seems to have a much deeper meaning of the “ultimate state of being” [161]. Often seen as a medical practice [162], yoga focuses on two main aspects: posture or asana; and regulated breathing, or “pranayama” [163]. Pranayama consists of (1) inhalation, (2) exhalation, (3) holding the breath after inhalation and (4) holding the breath after exhalation.

There are several types of pranayamas, and they all serve a particular intention [164]. The slow type of pranayama (3–6 breaths per minute) can significantly reduce heart rate [162]. Positive effects occur when the practitioner focuses on the energy (prana) flow through the seven chakras or a specific one to balance emotions or feelings. One of them, the solar plexus (“Manipura”) chakra, is located in the diaphragmatic region above the navel and the solar plexus, and it is attributed to willpower, opinions and beliefs, personal identity and decision-making. Practicing yoga (pranayama) or meditation combined with specific poses on a sunny day might help the practitioner find balance in the solar plexus chakra, stimulating the authentic self to shine brightly and increasing anti-inflammatory functions [165,166,167]. Beneficial effects of pranayamas on cardiovascular [162,163], autonomic [164], cognitive [168] and pulmonary functions [168] have been reported. While performing pranayama breathing techniques, participants are encouraged to focus on relaxation, which reduces anxiety [169]. Indeed, poor mental health has been associated with a higher risk of developing acute respiratory infection (Monto, 2002; Adam, Meinlschmidt and Lieb, 2013), which can be reduced with pranayama practice. Pranayama has significantly improved exercise tolerance in chronic obstructive pulmonary disease [169]. Improved functional capacity, lung function, mental health, and quality of life in patients point to pranayamas as an efficient alternative to other physical training activities and they might be a valuable tool to add to formal rehabilitation programs [170].

Yoga therapy might be used to aid in fighting COVID-19. Before affecting the lungs, the SARS-CoV-2 virus lodges in the throat region. The virus’s fatty acid coat adheres to the moist mucosal layers, binding to specific cell receptors and moving into the cells [171]. Yoga breathing techniques (pranayama) and postures (asanas) have a direct beneficial impact on the lungs and diaphragm [169,170], which can induce the innate immunologic response of the mucus membranes and may reduce the virus’ action in lung tissues [167]. Stress, anxiety and depression may increase the risk of acute respiratory infections, and, most likely, COVID-19 is no exception [171,172,173]. While reducing stress, anxiety, and depression, pranayama combined with meditation can promote lung function and virus-specific immune response, reducing inflammation bio-markers as cytokines activity [167,172,174,175,176]. Thus, just like Qigong and Tai Chi, yoga could be tested to improve the condition of COVID-19-recovering patients.

Yoga is another practice that focuses on simple breathing regulation to produce a specific functioning state. Yoga does share several aspects with Tai Chi and Qigong, such as the superconductors hypothesis, and the influence on the immune system, particularly on virus-specific responses.

## 4. Discussion

Homeostasis is a relevant concept in CM and CAM and is closely related to the human body’s integrity and the balance between the body, mind and the social and natural environment. Individual sensorial information is essential to analyze and evaluate the system’s dynamics, as external signs and perceptions differ [13,47].

The interaction between body, mind and consciousness is essential to maintain health. Balancing the body, mind and consciousness requires understanding how different forces (energies) work together [46,47,56,61,62,177]. Each person has a particular constitution and a specific energy pattern, an individual combination of physical, mental and emotional characteristics. This constitution is determined at conception and remains the same throughout life, even though internal and external factors might act upon us to disturb the balance. Western allopathic medicine focuses on symptomatology and disease and the primary use of drugs and surgery to rid the body of pathogens or diseased tissue, while homeopathic medicines focus on maintaining balanced energy flow and health.

CAM practices often use deep and specific breathing techniques to promote health manifestation. As previously seen, breathing influences labor experience in pregnant women in many positive ways. However, there is still little research on how different types of breathing can influence such a situation, and, consequently, there is a lack of evidence-based protocols worldwide.

Breathing diseases are strongly correlated with anxiety and depression [21]. Research suggests that specific breathing exercises can help improve asthma symptoms and chronic obstructive pulmonary disease (COPD) [113,135,178], which, by correlation, suggests that other breathing techniques might help improve symptoms of these conditions. If such disorders could be effectively treated using behavioral methods, expensive chemical treatments could be primarily replaced or reduced. The high correlation between these psychological and breathing-related disorders might even raise the question of which one is the “cause” of the other.

Indeed, the answer to part of the question raised in the last paragraph might be found in the studies conducted on diaphragmatic breathing [70], Qigong [103], Tai Chi [89] and yoga [179]. These show that breathing might increase parasympathetic tone instead of the sympathetic, reducing symptoms associated with psychopathologies.

It seems that oriental health practices have long emphasized breathing as a critical element of human health. Specific sets of respiration exercises have been developed and are, today, part of these systems. From specific Qigong exercises to pranayamas, there is a precise utilization of this mechanism as one of the main leverages for influencing physiology. In contrast, CM seems to make a non-systematized use of these physiological mechanisms. Breathing techniques are used in the labor room [66], in gastroenterology practice [69] or as a method to induce a relaxation response [74], but without a standard recommended use. Maybe for its simplicity, breathing has not been sufficiently explored and tested as a therapeutic method in CM. These practices commonly fall within the scope of CAM; therefore, due to politics and cultural resistance, CM does not highlight the importance of respiration regulation practices in wellbeing. Another reason for this undervaluation might be related to the costless character of these interventions, which makes it difficult to, on the one hand, believe in the potential benefits it has, and, on the other, to grant investment for further research. Regardless, a growing body of information points to the effectiveness of breathing workout practices in Qigong, yoga and Tai Chi. A systematic approach to the already existing breathing processes within CM is required. The integration of CAM breathing techniques mentioned in this work might be of value. Indeed, they do not aim to be alternatives but are complementary practices to promote wellbeing and cure diseases. Breathing seems to be one of many possible bridges between CAM and CM, potentially reducing the sometimes devastating side effects of long-term medication use. 

In the West, habits to promote and maintain health are often transmitted from generation to generation, from parents to sons and daughters. These include, but are not limited to, (i) choosing a healthy diet, (ii) maintaining personal hygiene, i.e., brushing one’s teeth, washing the hands and body, (iii) sleep hygiene, i.e., sleeping a certain number of hours per night, (iv) having an active lifestyle, i.e., practicing physical activity, and (v) adopting good postures (how to sit and stand up properly). It seems incomprehensible within this healthful culture that breathing habits, an essential element to promote health, have been left out. Considering the growing body of evidence pointing towards beneficial effects on health, respiration exercises should be further explored and integrated as part of every child’s basic teaching, whether in school, at home, or when visiting healthcare professionals. Breathing exercises should become a regular activity of everyday life. In this way, significant steps might be taken towards preventing debilitating conditions, i.e., anxiety, depression, and breathing disorders, in the youth and future generations. Western governments should evaluate the potential low-cost beneficial impact of proper breathing habits on public health. While incorporated into daily life, breathing exercises might positively affect human health if implemented as a standard routine in public health programs.

## 5. Conclusions

Breathing is a well-studied process in modern physiology with a high impact on health. Indeed, slight changes in the body’s chemistry induced by breathing impact all systems, including the immune system and its response. From a holistic point of view, the superconductor hypothesis of the ECM gains particular relevance. This deep and fast communication pathway might be closely related to the body’s self-regulation mechanism. Within TCAM, breathing is a central element. Practices such as Qigong, Tai Chi and yoga have been shown to help to reduce and prevent the effects of common diseases and might be powerful tools to promote lung function and virus-specific immune response. An in-depth systematic investigation of the benefits of such practices is encouraged to clarify their prophylactic character, especially considering the current COVID-19 pandemic.

Indeed, on 14 May 2020, Tedros Ghebreyesus, General Director of the WHO, said, “The impact of the pandemic on people’s mental health is already extremely concerning”. Furthermore, the United Nations highlighted the urgent need “to increase investment in mental health services who face to a massive increase in mental health conditions in the coming months” [180].

Systematic breathing training as a primary preventive health tool would be valuable to integrate into national health programs. It is easy to make it available to everyone; it is a low-cost intervention; it is safe and with no dangerous side effects; it is a non-stigmatized treatment complement (according to WHO, nearly two-thirds of the population with a known mental disorder never seek help from a health professional because of social stigma).

In the current pandemic, with increased mental distress and the risk of massive usage of community safe-guarding respiratory masks for long periods, implementing a daily routine of breathing workouts sounds like a reasonable preventive intervention. Furthermore, given the increased scientific attention in western medicine, integrating these practices as part of CM might be a turning point.

## Data Availability

Not applicable.
